# Mental Health and economic effects: correlation between unemployment and psychoactive drugs

**DOI:** 10.1192/j.eurpsy.2022.1619

**Published:** 2022-09-01

**Authors:** J. Ramos

**Affiliations:** Centro Hospitalar Cova da Beira, EPE, Departamento De Psiquiatria E Saúde Mental, Covilhã, Portugal

**Keywords:** mental health “add” economic crisis “add” psychoactive drugs

## Abstract

**Introduction:**

Mental Health is an invisible part of public health, and a determinant of it by affecting the human, social and economic capital of countries. Is one of the main causes of disability worldwide and, when left untreated, they can lead to increased costs and premature mortality. In 2019 they represented 22% of the disability burden in DALY in the European Union. Regarding the effects of economic recessions studies suggest that they have detrimental effects on mental health. And can became a reality in the current pandemic scenario.

**Objectives:**

To reflect on the studies carried out that debate the effects of previous economic crises on mental health, particularly the 2008-2013 crisis. It aims to list not only the possible intervention strategies in the area as well as the barriers to their implementation.

**Methods:**

Classic review of the topic through the international literature and the state of the art on available platforms. Establish a proxy between the unemployment rate and the number of packages (antidepressants and anxiolytics consumed) in homologous periods as a representative capacity of the impact of the crisis on mental health.

**Results:**

The number of packages of antidepressants and anxiolytics behave differently. the antidepressants have greater consumption when unemployment decreases.

**Conclusions:**

Several studies describe that the increase in the unemployment rate, indebtedness and social exclusion are empirically proven as consequences of the economic crises and predisposing factors for mental pathology. However, this does not translate into a proxy for the consumption of antidepressant packages with the increase in the unemployment rate. It may be due to the non-prioritization of mental health.

**Disclosure:**

No significant relationships.

